# Improved Cardiac Rehabilitation Referral Rate Utilizing a Multidisciplinary Quality Improvement Team

**DOI:** 10.7759/cureus.61157

**Published:** 2024-05-27

**Authors:** Cameron Whitler, Kyle S Varkoly, Harshil Patel, Andrew D Assaf, Jennifer Hoose, Grace D Brannan, Ronald Miller, Marcel Zughaib

**Affiliations:** 1 Department of Cardiovascular Medicine, Ascension Providence Hospital, Southfield, USA; 2 Department of Internal Medicine, McLaren Macomb Hospital, Mount Clemens, USA; 3 Department of Research, GDB Research and Statistical Consulting, Athens, USA

**Keywords:** multidisciplinary teams, management guideline, ischemic heart diseases, referral, cardiac rehabiliation

## Abstract

Introduction

Cardiac rehabilitation (CR) is an underutilized resource in patients with ischemic heart disease, despite being a Class IA recommendation. In this study, a multidisciplinary quality improvement (QI) team aimed to improve CR referrals by standardizing the ordering process at our hospital system.

Method

By using a collaborative approach involving the electronic medical record (EMR), medical provider education, and hospital protocols, our two-hospital healthcare system was able to successfully identify barriers to CR referral rates and implement interventions for these barriers. All physicians and medical providers, including ancillary staff, were educated on the EMR order sets to improve compliance by using automated order sets in the EMR. The CR referral order in the EMR included a statement regarding the application of evidence-based medicine, and a computerized provider order entry was included as a reminder to the ordering provider. The use of EMR was monitored monthly by the QI committee. Chi-square test and odds ratios were obtained for statistical analysis.

Results

Through provider-EMR education and patient education on discharge, CR referral rates significantly improved from 51.2 to 87.1% (p = 0.0001) in a 12-month period. The study included 1,499 patients in total. The improvement was statistically significant regardless of patient gender, race, or insurance coverage. Additionally, subgroup analysis in this study found that prior to standardization of the ordering process, African American patients were significantly less likely to be referred to CR compared to Caucasian patients. (51.2% vs. 41.0%, p=0.01). There was no statistically significant difference in the likelihood of CR referral between Caucasian and African American patients following the intervention (84.0% vs. 78.0%, p = 0.166).

Conclusion

This study shows that CR is an underutilized resource and that effective QI initiatives may not only increase CR referral rates but also close the gap between racial inequities in referral rates. Future research with multi-center randomized control trials is needed to further enhance its external generalizability to other institutions.

## Introduction

Cardiac rehabilitation (CR) is an evidence-based program proven to reduce morbidity, mortality, and hospital readmissions in patients with ischemic heart disease [1, 2]. Participation in a comprehensive CR program, a multidisciplinary program involving secondary cardiovascular risk reduction strategies, physical activity, health education, and stress management, is a Class IA recommendation in the 2021 American College of Cardiology (ACC)/American Heart Association (AHA)/Society for Cardiovascular Angiography & Interventions (SCAI) Coronary Revascularization Guidelines [3-4]. Despite this high level of recommendation, participation in CR continues to remain low [5]. Furthermore, certain subgroups, including ethnic minorities, women, and those with inadequate insurance coverage, have been shown to less likely to receive CR [5-13].

Starting in 2012 and renewed in 2017, the Center for Disease Control (CDC) and the Center for Medicaid and Medicare Services (CMS) started the “Million Hearts Campaign," which aimed to improve CR utilization with the goal of preventing one million cardiovascular events over five years by increasing CR participation from 20 to 70% [14]. Strategies employed by this campaign include monthly meetings amongst experts in CR invested in preventing a secondary cardiac event. Importantly, the campaign identified two essential components to increasing CR utilization: referral and enrollment.

Cardiac rehabilitation referral is defined as the combination of an order in the medical record with a discussion between the clinician and patient regarding CR participation and the receipt of the aforementioned order by a CR program. Previous studies have shown that systematic automated referrals, which are CR referrals triggered for eligible patients through an electronic medical record (EMR) once a stimulus is identified (i.e., certain diagnoses in the EMR), improve CR referral rates significantly [15]. However, despite prior attempts at the incorporation of EMR protocols, CR referral rates at our institution continued to remain relatively low. The reasons for this include a lack of interprofessional collaboration, leading to decreased systemic belief in CR effectiveness, as physicians were the sole drivers of CR referrals. The aim of this study was to evaluate the effectiveness of a multidisciplinary team’s collaborative approach. Past studies have done so using a similar Plan-Do-Study-Act (PDSA) methodology with success; however, their methodology was primarily fellow and resident-driven, whereas our methodology involves interprofessional collaboration while assessing the impact of healthcare disparities [16]. The objectives of this study were to evaluate the effectiveness of a multidisciplinary quality improvement intervention in increasing CR referral rates at hospital discharge and to assess the impact of the intervention on reducing racial disparities in CR referral rates.

## Materials and methods

This retrospective, observational study was performed at a two-hospital healthcare system, which is a large tertiary-level institution with a cardiovascular disease training program that performs more than 800 percutaneous coronary interventions (PCI) per year. One thousand four hundred and ninety-nine patients met the inclusion criteria within the allotted time frame of one year for pre-intervention and eight months for post-intervention. All patients ≥18 years of age who underwent PCI from January 2021 through December 2022 were included unless exclusion or exception criteria published by the National Cardiovascular Data Registry (NCDR) were met. These exclusion criteria included any patient with (1) comfort measures, (2) death during hospitalization, (3) left against medical advice, (4) were discharged to another acute care facility, or (5) were discharged with hospice care. Exception criteria included any patient with a medical or system reason(s) for not providing cardiac rehabilitation, as documented by a healthcare provider prior to hospital discharge.

Formulation of the quality improvement (QI) multidisciplinary team

A multidisciplinary QI team consisting of general cardiology fellows, interventional cardiology fellows, quality improvement nurses, cardiovascular program leaders, catheterization lab staff, and attending physicians was created. This collaborative, multidisciplinary effort was designed to investigate potential barriers to the CR referral rate and implement an interventional approach to improve CR referral rates. Members of the multidisciplinary team met monthly and performed a preliminary review of 52 randomly selected EMRs of patients who did not receive a CR referral on discharge.

The team carried out interventions for the following identified reasons.

Electronic Medical Record Utilization and Provider Education

During the patient chart audit period performed by the QI committee, the team learned that many providers were using outdated ordersets in the EMR, which did not trigger an automated alert at hospital discharge if a CR referral was not ordered (i.e., missed the “opt-out” mechanism in the EMR). As a result, physician education was provided on the following: 1) indications for CR referral; 2) how to appropriately document if CR was not desired; 3) how to use pre-existing EMR ordersets for management of post-PCI patients; 4) incorporation of an automated CR referral order in these ordersets; and 5) a new workflow for bedside nurses in the post-procedural care unit that would verify a CR order prior to patient discharge. Patient chart audits were then performed randomly by the QI committee to monitor EMR orderset utilization.

Patient Education and Hospital Discharge Protocol

In addition to incorrect EMR utilization, the QI committee identified other causes for missed CR referrals. These causes ranged from specialties other than cardiology discharging the patient, unexpected delays or other events in hospital discharge, or a lack of provider documentation for why a CR referral was not placed. Thus, the QI committee and nursing administration incorporated education on CR into the bedside nursing staff during routine meetings. Bedside nurses were encouraged to verify CR referral orders at patient discharge and were asked to contact the ordering physician if an order was missed. This allowed nurses to provide patient education on CR prior to hospital discharge.

Data Collection and Statistical Analysis

We collected pre- and post-intervention data. Baseline data on the CR referral rate were collected for 12 months, from January 2021 through December 2021. The intervention was conducted from January 2022 through February 2022. Patients during this period were not included in the final analysis to allow sufficient time for the interventions to take effect before assessing post-intervention outcomes. The interventions outlined above were instituted in April 2022 and continued through December 2022. Post-intervention data were collected starting in April 2022 through December 2022. Descriptive statistics such as means, frequencies, and percentages were generated. MedCalc statistical software (MedCalc Software Ltd., Ostend, Belgium) was used to compare cardiac referral rates pre- and post-intervention [16]. All other data were analyzed using the chi-square test and odds ratio using IBM SPSS Statistics software for Windows, version 29 (IBM Corp., Armonk, NY). Statistical significance was set at p <0.05.

## Results

A total of 1,499 patients were included in the analysis, with 896 patients in the pre-intervention cohort and 603 patients in the post-intervention cohort (Table 1). Patient cohorts were selected for analysis based on socioeconomic measures, including gender, race, and health insurance status, to determine if these had an impact on CR referral rates. Baseline demographic data revealed no significant differences in sex, race, age, insurance coverage, BMI, or medical comorbidities between the two cohorts.

**Table 1:** Patient demographics pre- and post-intervention PCI: percutaneous coronary intervention; CABG: coronary artery bypass grafting; ESRD: end-stage renal disease; BMI: body mass index; CSHA: Canadian Study of Health and Aging; N: sample size

Demographic information	Pre-intervention		Post-intervention	
	Patients (n)	(%)	Patients (n)	(%)
Total (n=1499)	896	59.8	603	40.2
Gender				
Female	275	30.7	184	30.5
Male	621	69.3	419	69.5
Race				
White	658	73.4	452	75
African American	198	22.1	115	19.1
American Indian	2	0.2	2	0.3
Asian	20	2.2	22	3.6
Unspecified	18	2	12	2
Health insurance				
No	10	1.1	1	0.2
Yes	886	98.9	602	99.8
Health insurance coverage				
Private only	376	42	206	34.2
Private and Medicare	336	37.5	159	26.4
Medicare	76	8.5	157	26
Medicare and Medicaid	25	2.8	24	4
Medicaid	48	5.4	42	7
State-specific	7	0.8	0	9
Other/Unspecified	28	3.1	15	2.5
Medical history				
Hypertension	796	88.8	540	89.6
Hyperlipidemia	793	88.5	525	87.1
Peripheral arterial disease	165	18.4	106	17.6
Chronic lung disease	131	14.6	99	16.4
Prior myocardial infarction	260	29	182	30.2
Prior PCI	395	44.1	271	44.9
Prior CABG	82	9.2	61	10.1
Prior tobacco abuse	521	58.1	355	58.9
Diabetes	404	45.1	271	44.9
ESRD	33	3.7	17	2.8
Arterial access site				
Radial	506	56.5	380	63
Femoral	390	43.5	223	37
Age in years (mean, in years)	66.27		67.19	
Time from PCI to discharge (average, in days)	1.42		1.23	
BMI (average, in kg/m^2^)	30.65		30.09	
CSHA Clinical Frailty Scale	4.23		3.99	

With regards to the baseline patient data, pre- and post-intervention, there was a statistically significant difference in the Canadian Study of Health and Aging (CSHA) Clinical Frailty Scale scores between the two groups (p = 0.001). There were no statistically significant differences in all other baseline variables between the two groups (the CSHA score ranges from one to nine).

**Figure 1:**
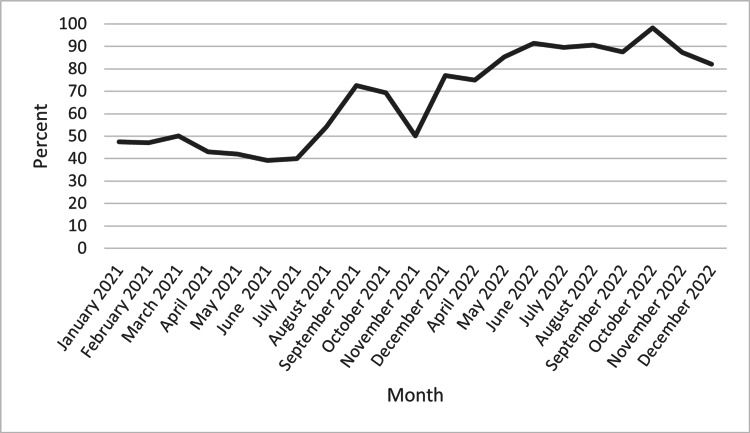
Cardiac rehabilitation referral rates by month, comparing pre-intervention (January to December 2021) with post-intervention (April 2022 to December 2022)

The pre-intervention cohort did have a significantly higher clinical frailty score (4.23 ± 1.014 vs. 3.99 ± 0.921, p<0.0001), defined by the CSHA score. Following the intervention, the CR referral rate amongst all eligible participants had a statistically significant improvement of 35.9%, increasing from 51.2% (n = 459/896) to 87.1% (n = 525/603) (p = 0.0001) (Table 2). Furthermore, the CR referral rate increased following the intervention, with a plateau at two months post-intervention and a peak at seven months post-intervention (Figure 1).

**Table 2:** Cardiac rehabilitation referral rate pre- and post-intervention *: Statistical significance was not achieved due to insufficient patient sample size for the designated cohort.

All patients (n=1499)	Pre-intervention (n=896)		Post-intervention (n=603)		p-value
	Patients (n)	(%)	Patients (n)	(%)	
	459	51.2	525	87.1	0.0001
						Gender					
Female	140	48.6	159	82.4	0.0001
Male	321	49.3	369	81.6	0.0001
Race					
White	352	51.2	400	83.7	0.0001
African American	86	41	98	78.4	0.0001
American Indian*	1	50	2	100	0.25
Asian*	11	52.4	18	72	0.17
Unspecified*	11	61.1	10	71.4	0.54
Health insurance coverage					
Private only	204	51.9	176	79.3	0.0001
Private and Medicare	159	45.7	141	83.4	0.0001
Medicare	44	55	139	84.2	0.0001
Medicare and Medicaid	10	38.5	20	74.1	0.0009
Medicaid	23	41.8	40	87	0.0001
Other or unspecified	16	53.3	12	75	0.0152

There was a significant increase in CR rates following the intervention. This increase was statistically significant regardless of patient gender, race, or insurance coverage. Patients of American, Indian, Asian, and unspecified races all had a trend toward improvement; however, this was not statistically significant due to the low cohort sample size.

The aforementioned interventions identified through the PDSA cycle by the QI committee resulted in an immediate and sustained improvement in CR referrals in the post-intervention cohort. In the first month post-intervention, the CR referral rate was 75.0%. The CR referral rate then ranged from 82.0% to 98.2% over the following months, with a total nine-month average of 87.1% (Table 1).

A statistically significant improvement in the CR referral rate in the post-intervention cohort was observed regardless of patient sex, race, or health insurance coverage (Table 2). The CR referral rate for women increased by 33.8% (48.6% (n = 140/288) to 82.4% (n = 159/193), p = 0.0001). The CR referral rate for men increased by 32.3% (49.3% (n = 321/651) to 81.6% (n = 369/452), p = 0.0001). In a subgroup analysis comparing the likelihood of CR referral between males and females, there was no statistically significant difference in pre- and post-intervention between men and women (Table 3).

**Table 3:** Pre- and post-intervention subgroup analysis comparing the likelihood of cardiac rehabilitation referral based on gender, race, and age

		Cardiac Rehab Referral Ordered					p-value
Demographic		Yes (n)	(%)	No (n)	(%)	Total (n)	
Gender							
Pre-intervention	Female	140	48.6	148	51	288	0.844
	Male	321	49.3	330	50.7	651	
Post-intervention	Female	159	82.4	34	17.6	193	0.822
	Male	369	81.6	83	18.4	452	
Race							
Pre-intervention	White	352	51.2	336	48.8	688	0.01
	African American	86	41	124	59	210	
Post-intervention	White	400	83.7	78	16.3	478	0.166
	African American	98	78.4	27	21.6	125	
Age							
Pre-intervention	<65 years	207	50.2	205	49.8	412	0.534
	≥65 years	254	48.2	273	51.8	527	
Post-intervention	<65 years	220	80	55	20	275	0.29
	≥65 years	308	83.2	62	16.8	370	

The CR referral rate for Caucasian patients increased by 32.5% (51.2% (n = 352/687) to 83.7% (n = 400/478), p = 0.0001, and the CR referral rate for African American patients improved by 37.0% (41.0% (n = 86/210) to 78.4% (n = 98/125), p = 0.0001). Pre-intervention, Caucasian patients were significantly more likely to be referred to CR when compared to African Americans: 51.2% (n = 352/687) vs. 41.0% (n = 86/210), respectively (p = 0.01) (Table 3). There was no statistically significant difference in the likelihood of CR referral between Caucasian and African American patients following the intervention (83.7% (n = 400/478) vs. 78.4% (n = 98/125), p = 0.166). At baseline, there were no significant differences in CR referral rates between varying types of insurance coverage; however, all subgroups of insurance coverage had a significant increase in CR referrals following the intervention.

Limitations in our study design include the retrospective observational nature of our study, as conclusions may not be drawn. Potential biases in data collection include information differential biases, such as recall bias, such as when the QI committee determined reasons for missing CR after contacting patients who missed an appointment opportunity.

A comparison of CR referral rates pre-intervention showed a statistically significant difference in referrals between Caucasian and African American patients. There were no differences identified between gender and age. Following the intervention, there were no differences in referral rates. The implications of the above findings suggest that once collaborative multidisciplinary interventions involving both physicians and ancillary staff are enacted, racial differences are absolved. Furthermore, similar community hospital settings may improve with the addition of evidence-based medicine and computerized provider order entry (CPOE) as CR referral orders in their respective EMRs, along with the incorporation of CR referral orders in the nursing discharge protocol.

## Discussion

In this quality improvement project, the CR referral rate increased from 51.2% (n = 459/896) to 87.1% (n = 525/603) over a 12-month period (p = 0.0001). In comparison to previous publications that examined the CR referral rates, our study had several important similarities and differences. First, in keeping with prior studies, the utilization of the EMR to trigger automated referrals can significantly improve the CR referral rate [15, 17-18]. A default or “opt-out” order for patients with qualifying diagnoses can result in efficient, systematic referral to outpatient CR during the hospital discharge process [16]. However, despite similar interventions at our facility in the past, the CR referral rate remained relatively low. Our QI committee identified optimization of the ordering process in the EMR as a key intervention during the PDSA cycle. Yet, a proportion of patients who missed referrals pre-intervention were not related to incorrect EMR orders. It was determined that a multi-level, collaborative approach would be necessary. Provider attitudes of physicians, nursing staff, and ancillary staff alike towards CR explained much of the low initial CR rate. By using a monthly QI meeting and changes to nursing discharge protocols, in addition to the utilization of the EMR ordering process with an evidence-based medicine reminder, our intervention resulted in immediate and sustained improvement in the CR referral rate over a nine-month observation period. Thus, our study shows that an automated EMR order alone may not be enough to improve the CR referral rate in some centers.

Increasing patient access to CR is an important goal. Cardiac rehabilitation carries the highest level of recommendation (Class IA) in the 2021 ACC/AHA/SCAI Coronary Revascularization Guidelines and has been shown to be a cost-effective intervention [5, 19-23]. For example, one systematic review found that all CR interventions were cost-effective, with incremental cost-effectiveness ratios ranging from $1,065 to $71,755 per quality-adjusted life-year (QALY), with varying numbers depending on the specific CR intervention (i.e., exercise, psychological counseling, telehealth visits) [24]. Clinician attitudes towards CR and inherent unconscious biases may be a reason for low CR referrals and utilization [1-13]. Improving physician and provider awareness has been shown to improve CR referral rates [19-21], and the strength of physician recommendation was an independent risk factor for CR participation in an observational study [19]. Efforts to improve awareness and standardize the ordering process to improve patient access are critical.

Our study also highlights racial disparities present in CR referral rates. At baseline, Caucasian patients in this study were significantly more likely to be referred to CR compared to African American patients (51.2% vs. 41.0%, p = 0.01). Our findings were consistent with those of other studies as well [6-10, 20-25]. A previous review of registry data for more than 48,000 patients found that African American patients were 20% less likely to receive CR referrals than Caucasian patients [22]. While inequity between Caucasian and African American patients was identified in the pre-intervention cohort in our study, importantly, this difference in CR referral rate was not present following our intervention (84.0% vs. 78.0%, p = 0.166).

This QI project is subject to limitations. Despite similar demographic characteristics between pre- and post-intervention cohorts overall, there was a significantly higher CSHA frailty score (i.e., “more frail”) in the pre-intervention cohort, which may have impacted the likelihood of referral. However, the authors feel that a higher frailty score in the baseline population does not impact the interpretation of the overall results. This study also examined CR referral orders at the time of hospital discharge and did not assess individual patient enrollment or completion of a CR program. Therefore, it is unknown if an increase in the CR referral rate increased overall CR enrollment. This could not be accurately tracked in this study given patients’ ability to enroll in CR at other hospital systems or third-party programs. Lastly, a study conducted in a single healthcare system is subject to inherent limitations regarding external validity. Therefore, further research on improving CR referral rates amongst the aforementioned cohorts in larger-scale studies involving multi-center studies is needed.

## Conclusions

In this quality improvement study, a multidisciplinary quality improvement team was able to establish an intervention that improved the CR referral rate by 35.9% (p = 0.0001) within a 12-month period. This collaborative intervention included optimization of the EMR ordering process, creation of a monthly QI meeting, and incorporation of nursing policies at hospital discharge. Retrospective analysis of the pre-intervention cohort revealed racial disparities in CR referrals, with Caucasian patients significantly more likely to be referred to CR compared to African American patients (51.2% vs. 41.0%, p = 0.01). This inequity was no longer present following the intervention. Closing the gap between racial disparities and CR referral rates was a key outcome of our intervention, and our proof-of-concept quality improvement study implicated this as being possible through multidisciplinary collaborative efforts. This study adds to previous literature that CR is an underutilized resource and that effective quality improvement initiatives can not only increase CR referral rates but also close the gap between racial inequities in CR referral rates. An additional strategy to improve cardiac rehabilitation healthcare access includes cost-effective home-based or community-based CR to further target an individual's unique health and socioeconomic needs.
